# Inclusion of Water Multipoles into the Implicit Solvation Framework Leads to Accuracy Gains

**DOI:** 10.1021/acs.jpcb.4c00254

**Published:** 2024-06-11

**Authors:** Igor S. Tolokh, Dan E. Folescu, Alexey V. Onufriev

**Affiliations:** † Department of Computer Science, Virginia Tech, Blacksburg, Virginia 24061, United States; ‡ Department of Mathematics, Virginia Tech, Blacksburg, Virginia 24061, United States; § Department of Physics, Virginia Tech, Blacksburg, Virginia 24061, United States; ∥ Center for Soft Matter and Biological Physics, Virginia Tech, Blacksburg, Virginia 24061, United States

## Abstract

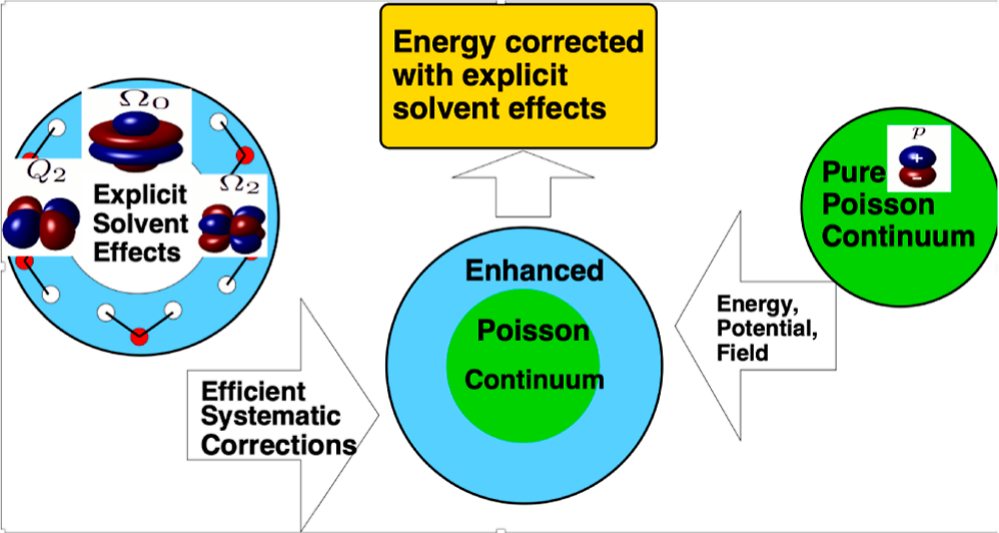

The current practical “workhorses” of the atomistic implicit solvation—the Poisson–Boltzmann (PB) and generalized Born (GB) models—face fundamental accuracy limitations. Here, we propose a computationally efficient implicit solvation framework, the Implicit Water Multipole GB (IWM-GB) model, that systematically incorporates the effects of multipole moments of water molecules in the first hydration shell of a solute, beyond the dipole water polarization already present at the PB/GB level. The framework explicitly accounts for coupling between polar and nonpolar contributions to the total solvation energy, which is missing from many implicit solvation models. An implementation of the framework, utilizing the GAFF force field and AM1-BCC atomic partial charges model, is parametrized and tested against the experimental hydration free energies of small molecules from the FreeSolv database. The resulting accuracy on the test set (RMSE ∼ 0.9 kcal/mol) is 12% better than that of the explicit solvation (TIP3P) treatment, which is orders of magnitude slower. We also find that the coupling between polar and nonpolar parts of the solvation free energy is essential to ensuring that several features of the IWM-GB model are physically meaningful, including the sign of the nonpolar contributions.

## 1 Introduction

Atomistic simulations are among the most widely used theoretical tools in biological research.^1−4^ High accuracy of the solvent representation in these simulations is paramount for biological applications, ranging from structure-based drug design^5^ to protein structure and p*K* prediction.^6,7^ Implicit solvation models treat solvents as a continuum with dielectric and nonpolar properties of water.^8−18^ Within the framework, the solvation effects are encapsulated by the total solvation free energy Δ*G*_solv_; an accurate and efficient estimation of this quantity as a function of the molecule’s conformation is critical for practical applications. Computational efficiency and throughput^19^ are principle advantages over the traditional, explicit solvent framework, though it should be noted that the implicit solvent approach is also well-suited to concise rationalization of observations via solid physical arguments.^20^

In general, both the polar (electrostatic) and nonpolar contributions to Δ*G*_solv_ need to be accounted for. The electrostatic effects^9,21−29^ are described by the electrostatic part of the total solvation free energy, Δ*G*_el_, which is often the most time-consuming computation in practical models. Accurate estimates of the total solvation energy, Δ*G*_solv_, and especially its change upon conformational transitions, require an accurate estimate of the nonpolar part,^5,30−33^ Δ*G*_np_. Coupling between Δ*G*_el_ and Δ*G*_np_, currently neglected by most practical implicit solvent models, can also be important^34−37^ when high levels of accuracy are required.

Within the implicit solvation framework, the Poisson Equation (PE) formalism^38−42^ is the “base line”, rigorous way to compute Δ*G*_el_. Along with its particularly computationally efficient approximation, the so-called generalized Born (GB) model,^13,31,43−72^ these two models are arguably the most widely used ways to compute Δ*G*_el_.^73−77^ Recent flavors of the GB model^32,78,79^ offer a level of accuracy closely approximating that of the numerical solutions of the PE in predicting solvation and binding free energies.

A hierarchy of approximations separates the PE/GB from the more fundamental explicit solvent models,^28,80−87^ and from reality,^15,88,89^ resulting in essential limitations such as the inability of the PE/GB to account for many prominent explicit solvent effects, such as charge hydration asymmetry (CHA)^90−101^ and other water multipole effects.^102−105^ The accuracy of Δ*G*_el_ within the PE/GB framework can be improved, to an extent, by adjusting atomic radii and increasing the number of atom types.^106−110^ Although efficient, traditional radii adjustments within the PE framework overfit for all of the missing physics, are poorly transferable,^111^ and do not deliver the level of accuracy of the explicit solvation. Models aiming to go beyond the popular PE for two dielectric media (solute cavity/solvent) consider various forms of position-dependent dielectric function,^112−118^ but still neglect important corrections from the multipole moments of water molecules beyond the dipole; these effects are also missing from several more sophisticated “beyond PE” solvent models based on point dipoles.^119−122^ Examples of other “beyond PE” models include RISM (3D-RISM),^123−126^ integral equation formalism,^127^ and explicit/implicit hybrid solvent models that consider the nearest to solute layers of solvent at the atomic level,^81,111,117,128−135^ including semiexplicit assembly methods.^136,137^ These models, useful in their respective domains, account for many of the explicit water effects “all at once”. Approaches based directly on the fundamental variational principles^34,36,107,115,138−141^ are arguably among the most conceptually advanced, physics-based implicit solvent models. Recently, approaches based on deep neural networks (DNNs) began to show promise in improving the accuracy of description of complex solvation effects,^142−149^ including a strategy in which the initial prediction by a physics-based implicit solvent model is further refined by a DNN correction.^150^

Currently, however, the implicit approach to solvation, as a whole, faces a unique challenge. Existing “workhorses” of fast implicit solvation models—the PE or GB—are no longer accurate enough for the increasing demands of the community in a growing number of areas, while sophisticated “beyond PE” models are still too slow to compete with conceptually simple, widely used explicit solvent models such as TIP3P.^151^ Yet, there is no fundamental reason why an implicit solvent model, with key physical effects accounted for and parametrized directly against experiment, should be less accurate than the explicit solvation, which is also an approximation to reality.

Here, we will explore an approach in which some of the key ingredients, present in the explicit solvent approach but missing from the PE and GB formalisms, are added to it as systematic corrections. The PE/GB is a physically well-grounded, accurate linear-response model at the macroscopic level, making it a natural “ground state” solution to which the missing microscopic effects can be added, one by one. Specifically, we will consider water multipole effects beyond the dipole already present at the PE/GB level of approximation. We will also explore the effect of an explicit coupling between polar and nonpolar contributions to the total solvation free energy, which is missing from common computationally efficient implicit solvation models. Since recent GB models^32,78,79^ offer a level of accuracy closely approximating that of the numerical PE, in what follows, we will use the computationally efficient GB as the “ground state” to which the proposed corrections are added.

## 2 Methods

### 2.1 Theoretical Background and Definitions

The total solvation free energy, Δ*G*_solv_, of a solute molecule in water consists of contributions from two mutually dependent, but different in their physical nature components: the polar/electrostatic, Δ*G*_el_, and nonpolar, Δ*G*_np_, free energies. The following two-step process is often used^88^ to define, rigorously and unambiguously, each of the components of the total Δ*G*_solv_. First, the nonpolar contribution to the total potential function, including the short-range repulsion and the attractive solute–solvent van der Waals dispersion terms, is gradually turned on in the absence of any long-range electrostatic interactions between the solute and the solvent. This step disrupts the water structure to create a cavity containing the molecule and the boundary separating the solute from the solvent; the corresponding reversible work is assigned to Δ*G*_np_, which is expected to be positive (unfavorable).^88^ During the second step, the solute–solvent electrostatic interactions are gradually turned on; the resulting reversible work is assigned to Δ*G*_el_. Note that the solute conformation is assumed to be fixed throughout the process. Since solvation of a charge inside a high dielectric medium^20^ is always favorable, Δ*G*_el_ < 0 in contrast to Δ*G*_np_. Furthermore, available experimental data for hydration free energies (HFEs) of small molecules^152^ indicate that Δ*G*_solv_ = Δ*G*_el_ + Δ*G*_np_ < 0 for the vast majority of realistic structures, which suggests that |Δ*G*_el_| > |Δ*G*_np_| in most cases; this expectation and that Δ*G*_np_ > 0 are confirmed by rigorous free energy calculations in the explicit solvent.^152^ Importantly, Δ*G*_el_ and Δ*G*_np_ are explicitly coupled; in particular, the position of the solute/solvent boundary, which affects the partition of the Δ*G*_solv_ into the electrostatic (polar) and nonpolar components, depends explicitly on both the electrostatic (e.g., the partial charges) and nonpolar (e.g., the Lennard-Jones (LJ)) parameters of the system. A clear manifestation of this coupling is the shift of the molecular boundary^34^ during the solute charging process—within the above framework, the corresponding component of the reversible work can be attributed to Δ*G*_el_. In implicit solvation models based on the variational principle, this coupling can be effected automatically, as the shape of the solute/solvent boundary is determined via the minimization of the appropriate free-energy functionals that depend on both the electrostatic and nonpolar terms.^34,37^

In contrast, in most practical, computationally efficient models based on the implicit solvation framework, Δ*G*_el_ and Δ*G*_np_ are computed separately and independently. These models typically start with defining and computing the geometry of the solute/solvent boundary, which, once constructed, remains fixed throughout the calculation (for the given conformation of the solute). Once the solute/solvent boundary is set, Δ*G*_el_ and Δ*G*_np_ are estimated independently of each other; the explicit coupling between Δ*G*_el_ and Δ*G*_np_ is not present.^a^

Arguably the simplest^88^ approximation for Δ*G*_np_ is to assume it to be directly proportional to the solvent accessible surface area (SASA) of the solute: Δ*G*_np_ = γ × SASA. More advanced approaches may include an explicit dependence on the LJ parameters describing the solute–solvent van der Waals dispersion interactions.^31,153^ Likewise, once the position of the solute/solvent boundary is set, multiple efficient approximations are available to compute the electrostatic component Δ*G*_el_. Physically, it originates from the interaction of the solute partial atomic charges, *q*_*i*_ at **r**_*i*_, with the solvent reaction field (RF) potential, ψ(**r**), induced by these charges. Within the common linear response approximation
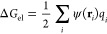
1

Once Δ*G*_el_ and Δ*G*_np_ are thus estimated, they are combined to yield
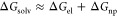
2

Note that now the resulting Δ*G*_solv_ is missing several physical effects, some of which are discussed below.

#### 2.1.1 The Missing Physics and an Outline of the Proposed Improvements

Within the implicit solvent framework, a common way to estimate ψ(**r**) is by using the formalism of continuum electrostatics, which includes the PE—a physically well-grounded, macroscopic level model, which considers a solvent as a dielectric continuum capable of dipole polarization in response to an applied electric field. The source of the applied field is the partial atomic charges of a solute surrounded by the solvent. Dipole polarization of the solvent induces surface charges at the boundary, separating the solvent and solute domains—the dielectric boundary (DB)^b^. In the framework of the PE model, which considers only dipole polarization of the solvent, the induced surface charges, described by a surface charge density (SCD) σ(**r**′) at the DB, are the only source of the solvent RF potential ψ(**r**).

However, when real water is the solvent, its molecules possess strong electric multipole moments higher than the dipole,^93^ and the polarization picture becomes more complex—quadrupole, octupole, and other higher multipoles have to be taken into account. These additional multipole components, missing from the “standard” PE and GB implicit models, can add significant contributions to ψ(**r**) and, therefore, to Δ*G*_el_.^156^

In what follows, we will introduce these “beyond the dipole” components, inherently present in the explicit solvent approach, into the formalism of continuum electrostatics. Considering the GB (or the PE) model as a natural “ground state” dipole level solution, the missing effects of the higher water multipoles, up to the octupoles, will be added here as separate, systematic corrections to the dipole term of the solvent RF potential.

We will also consider explicit corrections to Δ*G*_solv_ due to the coupling between the polar and nonpolar contributions present in the explicit solvent approach. This will be our model with the coupling (WC), in contrast to the baseline model with no explicit coupling (NC) between Δ*G*_el_ and Δ*G*_np_.

### 2.2 New Model, Δ*G*_el_: Water Multipole Corrections to the Reaction Field Potential

Our goal here is to move beyond the standard PE/GB formalism in computing the electrostatic (polar) component of the solvation free energy, eq 1.

We represent the solvent RF potential, ψ(**r**), as the sum of contributions from different spherical electric multipole moments of water molecules^98,103^ (up to the octupole): dipole (μ), square quadrupole (*Q*_2_), linear octupole (Ω_0_), and cubic octupole (Ω_2_). Due to the very small magnitude of the linear quadrupole (*Q*_0_),^103^ compared to *Q*_2_, its contribution is neglected. These water multipole contributions originate as a result of the orientational polarization of water layers around a solute, and can be expressed in the form of integrals over the DB around solute molecule
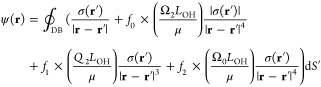
3

The *f*_*i*_ coefficients in eq 3 are dimensionless parameters, which are proportionality coefficients between the corresponding multipole polarization of water and the induced SCD σ(**r**′) due to the dipole polarization of water; *L*_OH_ is the OH bond length of the water molecule.

The *first* term in eq 3 corresponds to the “standard” PE/GB level of description related to the dipole polarization of the whole volume of the solvent, leading to the appearance of induced charges at the DB, as described by the SCD σ(**r**′). The *next three* “non-PE” terms are the corrections to the “standard” PE/GB description due to the higher multipole (quadrupole and octupole) polarization of water around the solute. Compared with the first dipole polarization term, in these higher multipole polarization terms, we neglect the contributions from all the layers of solvent around the solute beyond the nearest one (the first hydration shell). Such an approximation is justified because of the faster decays of the quadrupole (∼1/*r*^3^) and octupole (∼1/*r*^4^) RF potentials of the polarized solvent layers compared to a decay of the dipole RF potential (∼1/*r*^2^). As a partial compensation for the omission of these smaller multipole contributions from the subsequent layers of solvent, we use the DB as the position of the first layer of multipoles instead of the SAS—the position of the first hydration shell (the surface of the averaged positions of the water oxygen centers of the nearest to solute water molecules). This approximation also leads to only surface integration, as in the first term in eq 3. But, compared to the first dipole term, the induced SCD σ in these higher multipole terms is used as a measure of the corresponding local water multipole polarizations in the first hydration shell, which, as well as σ, in the linear response approximation are proportional to the E-field strength.

The *second* term in eq 3 is analogous to the charge-asymmetric part of the previously proposed charge-asymmetric Born formula^98^ and describes the CHA corrections to the RF potential. These corrections are proportional to the cubic octupole moment Ω_2_ of the water molecule,^98,157^ recognized^93,98,103,158^ as key to CHA effects, and should be independent of the sign of the dipole polarization represented by σ(**r**′). The cubic octupole moment is defined as Ω_2_ = (1/2)(Ω_*yyz*_ – Ω_*xxz*_), where Ω_*ijk*_ are the components of the traceless octupole tensor of the water molecule charge distribution in the standard Cartesian frame for water molecules.^159,160^

The *next two* terms represent the corrections to the RF potential due to the charge symmetric contributions of the planar quadrupole *Q*_2_ = (1/2)(*Q*_*yy*_ – *Q*_*xx*_) and linear octupole Ω_0_ = Ω_*zzz*_ moments of water molecules.

Due to the absence of experimental values for the quadrupole and octupole moments of water molecules in water and some differences between their quantum mechanical estimates and their explicit water model values,^103,161^ we combine the scaling coefficients *f*_*i*_ and the multipole moment values in eq 3 into optimizable parameters *F*_*i*_ (*i* = 0, 1, 2), and rewrite eq 3 in the form
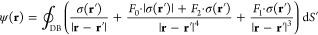
4where  and . The last two terms in eq 4 represent multipole corrections to the RF, contributions of which to the hydration energies are calculated by eq 11.

While the above equation is mathematically equivalent to eq 3, it has an advantage in terms of computational efficiency: only two additional surface integrals need to be computed on top of the dipole polarization part represented by the first term. This means that, theoretically, the computational cost of the new model can be only about three times the cost of the standard GB, which corresponds to the first term. Our in-house unoptimized scripts, that compute the last two terms in eq 4, are about ten times slower compared to the optimized Cartesian grid-based surface GB (GBNSR6) code^78,162,163^ in AmberTools, used here to estimate the contribution of the first term to Δ*G*_el_; we believe that these costs can be reduced based on the argument above.

### 2.3 New Model: Nonpolar Interactions and Coupling between Δ*G*_np_ and Δ*G*_el_

As the baseline approximation for Δ*G*_np_, we use the approach of Gallicchio et al.,^31,153^ which combines the surface tension term (cavity term) with one that depends explicitly on van der Waals dispersion interactions between the solute and the solvent
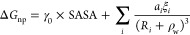
5where γ_0_ is the (constant) surface tension parameter, SASA is the solvent accessible surface area, *a*_*i*_ is the product^163^ of the water number density and the LJ interaction parameters of a solute atom *i* with TIP3P water oxygen atom, ξ_*i*_ is the scaling factor that can depend on the atom type, *R*_*i*_ is the *i*-th atom’s “R6” effective Born radius,^68,163−165^ and ρ_w_ is the water probe radius. This is our no coupling (NC) model.

However, as discussed in Section 2.1, the correct physics of aqueous solvation includes a coupling between Δ*G*_np_ and Δ*G*_el_ contributions to the total HFE. We introduce this polar/nonpolar coupling in an efficient, albeit approximate, manner. We again utilize the approximation in which the solute/solvent boundary is fixed at the start, use the nonpolar terms proposed by Gallicchio et al.,^31,153^ but introduce an explicit dependence of their key parameters on the solute E-field. Thus, the surface tension, γ = γ_0_, in the cavity term in eq 5 and the effective distance between the DB and SAS, *D* = ρ_w_, in the van der Waals dispersion interaction term in eq 5 become explicitly dependent on the solute E-field. Considering that the strength of E-field at the DB or SAS is proportional to corresponding local σ(**r**) values, we propose the following implicit water multipoles (IWM)-GB model with an explicit coupling (WC) between polar and nonpolar contributions
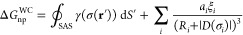
6

Here, γ(σ(**r**′)) is the surface tension parameter, which now depends explicitly on the E-field strength at point **r**′ at SAS; this E-field strength is proportional to a formal quantity, σ(**r**′), which is a “SCD” calculated at SAS, used here as a measure of the E-field strength at SAS; and distance *D*(σ_*i*_), is the effective distance separating the DB and SAS, near the solute atom *i*. In the IWM-GB WC model, *D*(σ_*i*_) depends explicitly on the solvent dipole polarization near atom *i*; in the model, the latter is expressed through the average SCD σ_*i*_ localized on the fraction of the *i*-th atom’s surface area^31^ of the DB.

Table 1 presents the two functional forms considered for polar/nonpolar coupling variants for the IWM-GB models: NC and with coupling (WC). Note that the coupling-dependent functions γ(σ(**r**′)) and *D*(σ_*i*_) must be even functions of σ(**r**′) (approximating the E-field at SAS) and σ_*i*_ (calculated at the DB), since, to the first approximation, the shift of SAS or change of γ(σ(**r**′)) due to a change in σ are independent of the sign of σ (direction of E-field at **r**′ on the SAS or at the DB near the solute atom *i*). Also note that, despite the fact that the function describing the coupling is introduced into the term that accounts for the nonpolar contribution, eq 6, the coupling itself is electrostatic in its origin, as it should be (see Section 2.1). Indeed, the magnitude of the coupling,  for *q*_*i*_ → 0, as is the case for Δ*G*_el_.

**Table 1:** Polar/Non-Polar Coupling Functional Forms for γ(**r**′) and *D*(σ_*i*_) in eq 6^a^

coupling variant	γ(σ(**r**′))	*D*(σ_*i*_)
*none* (NC)	γ_0_	ρ_w_
*quartic* (WC)	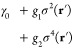	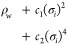

a Within the two coupling variants (WC or NC), the optimized parameters include: the global surface tension γ_0_; the cavity portion coupling coefficients *g*_1,2_; and the van der Waals portion coupling coefficients *c*_1,2_.

After adding four coupling parameters to the IWM-GB WC model, to keep the size of the parameter space of this model similar to that one of the IWM-GB NC model, we have reduced the number of dispersion interaction scaling factors, ξ_*i*_, from four (in the IWM-GB NC model) to just one, making it solute atom type independent. Such a parameter reduction step allows us to have only one extra parameter in the IWM-GB WC model compared to the IWM-GB NC model.

### 2.4 Optimal Dielectric Boundary

**Figure 1:**
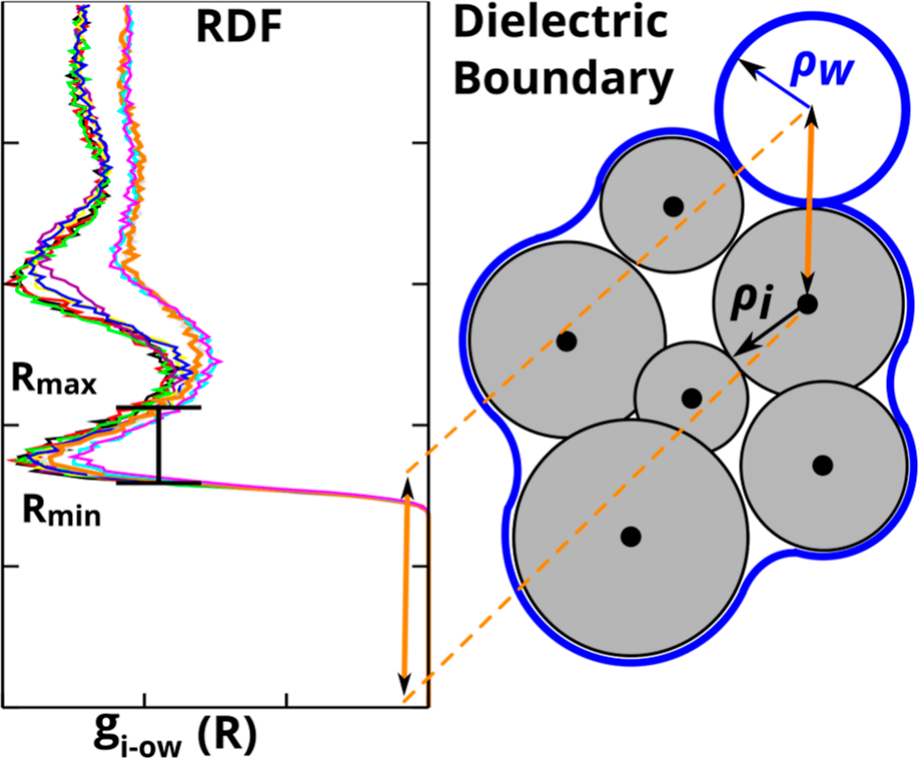
Optimal DB based on the “solute atom”–“water oxygen” radial distrtibution function (RDF) physical constraints. Water probe radius, ρ_w_, and intrinsic atomic radii, ρ_i_, are optimized simultaneously, under physically justified constraints that ρ_i_ + ρ_w_ is bounded within the first peak of the RDF, between *R*_min_ and *R*_max_. RDFs were computed in the explicit solvent for a diverse set of biomolecular structures.^166^

Just like in the standard PB treatment, in our approach, the DB, represented as a solvent excluded surface (SES), has a fixed position, which we optimize under a set of physical constraints, Figure 1. Note that even with a fixed DB geometry, the important physics of solvation does not have to be missing from the computed solvation free energy—it can be accounted for through additional energy corrections to the basic PE/GB level of description of polar/electrostatic interactions in Δ*G*_solv_. Fixing the DB^c^ offers clear computational advantages that are important for the satisfactory performance of our optimization procedure.

For each optimization of model solvation free energies, we have ensured that constructed DBs obey physically well-grounded and experimentally observed constraints: specifically, optimization of intrinsic atomic radii (ρ_i_) and water probe radius (ρ_w_) were completed such that, for each solute atom type, the distance from the solute atom to water oxygen was constrained to the first peak of the water oxygen radial distribution function (RDF), Figure 1. This constraint alone mitigates model overfitting concerns, and has allowed for a more comprehensive exploration of boundary representations with physically reasonable radii values. In addition, to limit computational complexity and, in effect, enhance the *transferability* of our proposed models, radii explorations have been limited to a minimal set of solute atom types, as detailed further in Section 2.6.2.

### 2.5 Numerical Implementation

#### 2.5.1 Dielectric Boundary Representation

We define the DB around solute molecules to be the SES and represent it as a triangular mesh, computed using the open-source NanoShaper package.^167^ This representation is well suited for the purpose of calculating apparent SCD σ and subsequently derived IWM corrections. PQR and XYZR files are generated from the topology and coordinate files of the corresponding solute molecule using the *ambpdb*(168) utility from the AMBER package. In each triangulation, a grid size of 0.5 Å is used. We have performed a limited exploration of the influence of this grid size on the speed and convergence of the whole pipeline for computing Δ*G*_solv_ (not including the optimization). Reducing the NanoShaper grid size from 0.5 to 0.25 Å results in about 7-fold increase in the computational time to obtain Δ*G*_solv_: the corresponding root-mean-square (RMS) deviation of Δ*G*_solv_ over the train set between the 0.25 and 0.5 Å computations is 0.23 kcal/mol, which means that the calculation at 0.5 Å is already reasonably converged, while further the putative reduction of the grid size to 0.25 Å is not justified by the increase of the computational time. On the other hand, increasing the grid size to 0.75 Å results in RMS deviation of 0.57 kcal/mol relative to 0.25 Å grid; the corresponding computational time is reduced by slightly less than a factor of 2 compared to the 0.5 Å computation.

#### 2.5.2 Computation of Surface Charge Densities

We utilize a fast, analytical formulation for the apparent SCD,^169^ based on the general approach to analytical electrostatics of biomolecules.^170,171^ For any point **r**′ on our DB representation, the apparent SCD at **r**′ is calculated as
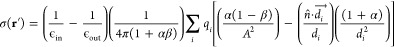
7where ϵ_in_ and ϵ_out_ are the dielectric constants of the solute and the solvent, α = 0.580127,^172^ β = ϵ_in_/ϵ_out_, *q*_*i*_ is the partial atomic charge of the solute atom *i*,  is the outward facing surface normal at the surface patch containing **r**′,  is the vector from the solute charge *q*_*i*_ to the aforementioned surface patch, and *A* is the so-called electrostatic size of the solute,^172,173^ which characterizes its global shape; physically, it provides a general connection between the solute’s overall shape/size and its electrostatic energy, see ref (173) for the exact definition. The quantity can be estimated efficiently and analytically by approximating the solute as an ellipsoid;^173^ for a spherical solute, *A* is simply its radius.

The accuracy of the analytical formulation, eq 7, is comparable to the standard PE treatment,^169^ but the calculation speed is orders of magnitude faster, as expected from an analytical treatment of electrostatics.^169,171^

For any molecule and the corresponding DB mesh, the SCD σ_*T*_, associated with a triangle *T* on the mesh, was computed by averaging the SCD values at its constituent vertices (computed via eq 7). The average SCD on the DB fraction of the *i*-th atom’s surface area, σ_*i*_, was computed as follows: first, we associate each triangle *T* with its *nearest atom*, which we define to be the atom that minimizes the distance between its center and the first triangle *T* vertex (numbering of triangle vertices and computation of the nearest atom to all vertices is a byproduct of triangulation via NanoShaper^167^); then, with the set of triangles on the mesh nearest to the *i*-th atom denoted by , we put
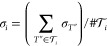
8where  denotes the number of triangles in the set .

#### 2.5.3 Computation of Standard Polar Solvation Energy (Water Dipole Level Approximation)

The standard approximation in calculating the polar/electrostatic component (Δ*G*_el_) of the total HFE of a molecule is to consider the polarization of water around the solute only at the dipole level. This approximation corresponds to the first term in eq 3 above for the solvent RF. While numerical PE is often used for this purpose, here we compute the contribution of this dipole RF term to the Δ*G*_el_ using the GBNSR6 code,^78,162,163^ available in the AMBER package. The GBNSR6 model closely approximates^78,79^ the numerical Poisson treatment in predicting solvation and binding free energies while offering a distinct computational advantage that becomes critical for multidimensional optimization of the model parameters. In the GBNSR6 method,^162,163^ the dipole level Δ*G*_el_^dipole^ can be calculated using the analytical linearized Poisson–Boltzmann (ALPB) approach,^172,173^ which introduces physically correct dependence on the dielectric constants ϵ_in_ and ϵ_out_ into the original GB model of Still et al.^47^ while still maintaining the efficiency of the original. We utilize the ALPB model, where the dipole level contribution, Δ*G*_el_^dipole^, to the total HFE (in the absence of salt in solution) is calculated as
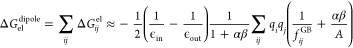
9

Here, , where *R*_*i*_ and *R*_*j*_ are the effective Born radii^47^ of atoms *i* and *j*, and *r*_*ij*_ is the distance between atomic charges *q*_*i*_ and *q*_*j*_. The inclusion of the salt effects into the ALPB approach^173^ is described in the following subsection. The GBNSR6-based calculations were performed at the 0.25 Å Cartesian grid spacing. Inner and outer dielectric constants were ϵ_*in*_ = 1 and ϵ_*out*_ = 80, respectively. All other parameters were set to their GBNSR6 default values (including a uniform correction^165^ to *R*_i_^–1^, *B* = 0.028 Å^–1^).

#### 2.5.4 Screening Effects of Mobile Ions

In the presence of salt in the solvent, the screening effects of mobile ions can be included into the “standard” dipole level contribution to the polar hydration energy Δ*G*_el_^dipole^ (eq 9) at the PB/GB mean-field level.^16,60^ By construction, within our new models, the correction to the HFE due to the salt screening is exactly the same as in the base GB theory. This is achieved by replacing
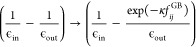
10in eq 9, which is employed by all our IWM-GB models. Here, κ is the inverse Debye length determined by the ionic strength of the solvent. The effects of salt at this level of theory have been well-tested in the past,^41,60,107^ and, thus, we do not pursue further testing of these effects here. During parameter optimization of our proposed IWM-GB models, we consider the ion-free solvent around a solute molecule (κ = 0) and choose the corresponding experimental reference data accordingly.

#### 2.5.5 Computation of Higher Water Multipoles Corrections and Nonpolar Energy

Continuous surface integrals, specified in Section 2.2, are computed using discretized sums over triangular surface elements of the DB. Orientational polarization of the higher multipole moments of water molecules around a solute creates additional (beyond dipole) components, ψ^corr^(**r**), of the solvent RF potential, which are specified by the second and third terms in eq 4. To calculate corrections to the “standard” (dipole polarization level) electrostatic HFE (Δ*G*_el_^dipole^, eq 9) due to these additional higher multipole components, we discretize the electrostatic energy correction, (Δ*G*_el_^corr^, as
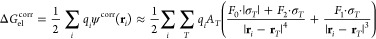
11where *A*_*T*_ and σ_*T*_ are the area and SCD of the triangle *T* on the DB, |**r**_*i*_ – **r**_*T*_| is the distance between the *i*-th atom center and the centroid of *T*, and *F*_0_, *F*_1_, and *F*_2_ are the optimization parameters corresponding to higher water molecule multipole moments (see eq 4). Once Δ*G*_el_^corr^ is estimated, the total electrostatic HFE can now be expressed as Δ*G*_el_ = Δ*G*_el_^dipole^ + Δ*G*_el_^corr^.

To calculate the nonpolar component Δ*G*_np_ (eqs 5 and 6) of Δ*G*_solv_, it has been discretized as
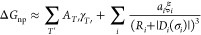
12where *A*_*T*′_ is the area of the triangle *T*′ on the SAS; γ_*T*′_ is the surface tension parameter, which can depend on the electric field strength at *T*′ in the case of polar/nonpolar coupling (see Table 1) or be constant; *a*_*i*_ is the product^163^ of the water number density and the LJ interaction parameters of the *i*-th solute atom with a water oxygen atom (we use TIP3P water oxygen and the AMBER 8 force field solute atoms LJ parameters); ξ_*i*_ is a scaling factor (subject to optimization) that can depend on the elemental type of the *i*-th atom;^163^*R*_*i*_ is the effective Born radius^47^ of the *i*-th atom; and *D*_*i*_(σ_*i*_) is the effective distance separating the DB near the solute atom *i* and SAS; *D*_*i*_ can be a constant (*D*_*i*_ = ρ_w_) or, in the case of the IWM-GB WC model, can depend on the average SCD σ_*i*_ located on the DB fraction of the *i*-th atom’s surface area. The functional forms of γ_*T*′_ and *D*_*i*_(σ_*i*_) are given in Table 1. To simplify the calculation of γ_*T*′_ in the case of the IWM-GB WC model, as a measure of the E-field strength at SAS, we use formal values of “surface charge densities” σ(*T*′) calculated at SAS using eq 7. We use the absolute value of *D*_*i*_(σ_*i*_) to prevent a nonphysical decrease in the denominator of the second term of eq 12, which would place the effective position of SAS below the DB.

### 2.6 Optimization

#### 2.6.1 Datasets

We have utilized a pared-down version of the set of 248 rigid neutral small molecules, initially selected by Mukhopadhyay et al.^174^ from a set of 504 neutral small organic molecules.^86^ In that selected set, the molecules were classified as rigid—having a very small conformational variety under molecular dynamic (MD) simulations.^174^ From that set of rigid molecules, we have further selected 173 molecules, which include only hydrogen, oxygen, nitrogen, and carbon atom types, and used them in the train (88 molecules) and test (85 molecules) subsets for the model optimization. The topology and coordinate files as well as experimental and explicit solvent HFEs were taken from version 0.52 of the FreeSolv database,^152^ with the updates and corrections indicated in ref (175). The selection of rigid molecules mitigates conformational sampling errors and reduces variation in calculated solvation effects.

To test the performance of the IWM-GB models on different types of molecules, we used 44 conformations (two conformations per molecule) of 22 blocked amino-acids (AA) and the corresponding TIP3P polar parts of their HFEs.^109,174^ In addition to standard 20 blocked AA,^109^ two neutral forms (due to protonation) of charged Glu and Asp blocked AA (Glh and Ash)^174^ are also considered.

#### 2.6.2 Overview

**Figure 2:**
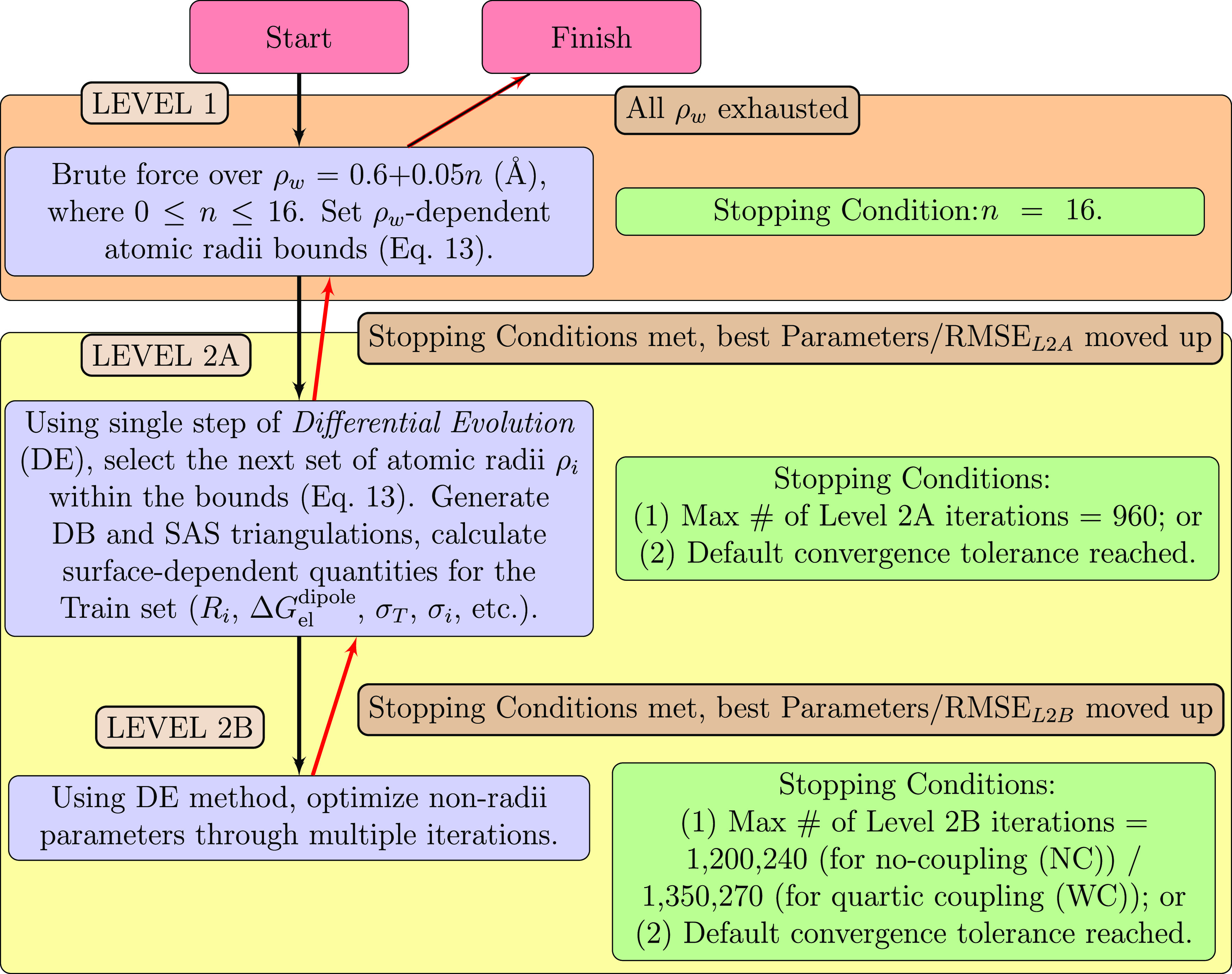
Three interconnected levels comprise the full model parameter optimization procedure; the objective function that is minimized, is the RMS error (RMSE) between IWM-GB model hydration free energies (Δ*G*_solv_) and the experimental references for the train set of molecules. Discrete water probe radius values are assigned in Level 1. Optimization of the remaining radii and nonradii parameters in Levels 2A and 2B, respectively, is made by distinct instantiations of the *Differential Evolution* (DE) optimization method.^176,177^ For each instantiation, a collection of full model parameter sets and their corresponding RMSEs is retained, from which the best full model parameter set at the current iteration is defined to be the set in the collection, which yields the lowest RMSE on the train set. Subsequent parameter search directions in Levels 2A and 2B are determined by the radii and nonradii subsets of the best full model parameter set at the current iteration. In Level 1, the water probe radius ρ_w_ is specified as {0.6 (Å) + 0.05*n* (Å), 0 ≤ *n* ≤ 16}; for each of these values, ρ_w_-dependent atomic radii bounds are determined in eq 13. Within these bounds, using the DE method, a set of atomic radii is selected in Level 2A to generate DB and SAS triangulations for the train molecule set and to compute related quantities, such as effective Born radii or SCDs, used in eqs 9, 11, and 12. In Level 2B, using the DE method, nonradii parameters are selected within the specified parameter bounds, see eq 14. At this stage, the radii and nonradii parameters define a *Full model parameter set*, for which Δ*G*_solv_ energies are computed and the corresponding RMSE to the reference is determined.

The overall optimization procedure consists of three “Levels” of manipulation and optimization of model parameters (see Figure 2): a brute force search over the water probe radius (Level 1), whereas the next two nested levels of optimization (Levels 2A and 2B) are distinguished via the partition of computationally cohesive model parameters; these nested levels reflect a grouping of the model parameters into disjoint “molecule intrinsic” (Level 2A) and “molecule extrinsic” (Level 2B) subsets. This partitioning was necessitated by the large difference in absolute computational time needed to choose and utilize the respective parameter subsets toward objective function evaluations. Such a splitting allows for a better exploration of the model parameter space, once the computationally heavy data generation steps in Level 2A (calculation of the *R*_*i*_ and Δ*G*_el_^dipole^, DB and SAS triangulations, SCD calculation, etc.) are completed.

We summarize our optimization workflow as follows: for a particular choice of water probe radius (Level 1), the bounds for atomic radii (see eq 13) are set. These bounds proposed in ref (166) were originally formulated using the deviations about the mean position of the first peak of the RDF of the water oxygen, which include the size of the underlying water probe radius. In turn, sets of atomic radii (molecule intrinsic parameters, Level 2A) can be chosen, and associated data generation (the computationally heavy portion of the optimization procedure) can be undertaken. Using these data, molecule extrinsic parameters can be explored in Level 2B by evaluating model functionals (now completely determined by the intrinsic and extrinsic parameters) that yield full HFEs for molecules comprising the dataset. For each complete set of model parameters, the RMS error (RMSE) between model derived and reference HFEs (unless otherwise stated, the experimental HFEs are used as the reference) is estimated. The RMSE for the train set of molecules (see Section 2.6.1) is used as an objective function in the exploration and optimization of molecule intrinsic and extrinsic parameters in Levels 2A and 2B by applying the *Differential Evolution* (DE) method.^176−178^ We designate a set of parameters with the smallest RMSE, possibly with additional constraints described below, as an *optimal* choice of model parameters (over the set of molecules on which we compute the RMSE, see Section 2.6.1).

#### 2.6.3 Details

In Level 1, a brute force iteration over 17 discrete values of the water probe radius ρ_w_ is undertaken, see Figure 2. Once the value of ρ_w_ is chosen, we determine ρ_w_-dependent upper bounds on the atomic radii of individual solute atom types, to be used in Levels 2A and 2B, as
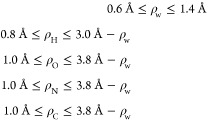
13

The bounds in eq 13 are restricted versions of the physically justified bounds, as discussed in ref (166). Notably, lower bounds for ρ_w_ and individual atomic radii (except hydrogen) have been set to 0.6 and 1.0 Å, respectively, as compared to 0.2 and 0.6 Å, respectively, in ref (166). These additional restrictions on the radii bounds were due to observed instabilities in the construction of the fixed DB/SAS representations (via NanoShaper) using very small, nonstandard radii selections.

In Level 2A, the initial atomic radii are randomly selected within the determined bounds (eq 13), whence DB and SAS representations for the train set of molecules are generated. In Level 2B, the initial nonradii parameters of the model are randomly selected using the lower and upper bounds in eq 14. These include polar energy multipole correction parameters *F*_0_, *F*_1_, and *F*_2_ (see eq 11), nonpolar energy parameters γ_0_ and ξ_*i*_ (see eq 12), and polar/nonpolar coupling parameters *g*_*j*_ and *c*_*j*_ (see Table 1).
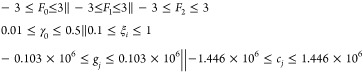
14

The absolute values of the bounds were chosen based on the maximum expected values of the corresponding parameters.

Having a complete set of model parameters selected, the IWM-GB model HFEs for the train set of molecules are computed in Level 2B, and the RMSE for these energies relative to the experimental references is calculated. The sets of best RMSEs and the corresponding values of the parameters are used to guide the parameter search directions (within each Levels 2A and 2B) in the subsequent iterations.

Throughout the selection and optimization procedures in Levels 2A and 2B (see Figure 2), we utilize the DE global optimizer from the *SciPy* optimization package.^177,178^ Level 2A featured 15 evolved generations (Gen_2*A*_) with a population size of 15 (Pop_2*A*_). Level 2B featured 5000 evolved generations (Gen_2*B*_) with a population size of 30 (Pop_2*B*_). The maximum number of iterations, maxIter_*j*_, at Level *j* (*j* = 2*A*, 2*B*) corresponds to the maximum number of function evaluations (without polishing) estimated as a product of the following parameters
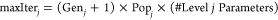


The optimization procedure is performed in parallel using no more than 128 GB of RAM on dual-socket AMD EPYC servers, furnished by ARC (https://arc.vt.edu) and CSRVM (https://csrvm.cs.vt.edu) at Virginia Tech.

For each proposed IWM-GB model functional form (NC and WC), optimization in Level 2B was performed with independent, fixed starting seeds. With parameters given as above, optimization for each choice of ρ_w_ (selected at Level 1) was completed in less than 2 days per run. With 17 ρ_w_ values, each optimization of a proposed model functional form was completed in less than 26 days of total computational time.

#### 2.6.4 Optimized Sets of Atomic Radii and Nonradii Parameters of the IWM-GB Models

The optimized coupling parameters *c*_1_ and *c*_2_ for the IWM-GB WC model lead to a reasonable value for the change, in response to “turning on” the atom’s electric field, of the effective distance *D*_*i*_(σ_*i*_) separating the DB and SAS^31^ near the solute atom *i*. For example, the estimated change of *D*_*i*_(σ_*i*_) (calculated as ) for the solute oxygen (O) atom with one of the largest negative partial charge (−0.6e) is about −0.3 Å. Another pair of optimized coupling parameters *g*_1_ and *g*_2_ lead to an increase of the effective surface tention γ(σ(**r**′)) at a portion of the SAS closest to the solute oxygen atom from 0.04 kcal/mol/Å^2^ (for an uncharged atom; σ(**r**′) = 0) to 0.16 kcal/mol/Å^2^. This range of γ is in a reasonable agreement with the value of γ = 0.117 kcal/mol/Å^2^ proposed by Gallicchio and Levy^31^ for the AGBNP implicit solvation model.

## 3 Results and Discussion

### 3.1 Accuracy of the IWM-GB Models

Here, we present the results of optimization and testing of our new IWM-GB models, introduced in Sections 2.2 and 2.3, and compare them with other solvation models.

Table 4 shows the main result—the performance^d^ of our two optimized IWM-GB models; the corresponding optimized parameters can be found in Tables 2 and 3. The proposed models demonstrate clear improvements in accuracy, measured via the RMSE against experiment, over the train and test sets of small molecules, as compared to the often-used explicit TIP3P water model. Both NC and WC variants of our IWM-GB model have seen an improvement of average RMSE (over the two sets) of ∼22%, as compared to the explicit solvent (TIP3P) results. The RMSE improvements over only the test set are ∼19 and ∼12% for the NC and WC variants of the model, respectively.

**Table 2:** Optimized Atomic Radii for the IWM-GB Models: With Coupling (WC), “No-Coupling” (NC), and WC “Balanced” Solution^a^

model	atomic radii (Å)				
	ρ_H_	ρ_O_	ρ_N_	ρ_C_	ρ_W_
IWM-GB WC	1.26	1.12	1.18	1.95	0.70
IWM-GB NC	0.82	1.02	1.57	1.63	0.80
IWM-GB WC balanced	1.20	1.01	1.09	1.90	0.90
BONDI	1.20	1.52	1.55	1.70	1.40
ZAP-9	1.10	1.76	1.40	1.87	1.40

a Standard Bondi^179^ and ZAP-9^110^ atomic radii, used in some of our calculations, are also shown for reference.

**Table 3:** Optimized Non-Radii Parameters Used in Eqs 11 and 12, and Eqs in Table 1 for the IWM-GB Models without Polar-To-Nonpolar Coupling (NC, Left Panel) and With Coupling (WC) and WC “Balanced” Solution (Right Panel)

	IWM-GB model
parameter	NC
*F*_0_ [Å^3^]	5.788 × 10^−2^
*F*_1_ [Å^2^]	–1.452
*F*_2_ [Å^3^]	9.528 × 10^−1^
γ_0_ [kcal/mol/Å^2^]	4.017 × 10^−2^
ξ_H_	6.802 × 10^−2^
ξ_O_	5.000 × 10^−2^
ξ_N_	5.981 × 10^−1^
ξ_C_	1.254 × 10^−1^

IWM-GB models		
parameter	WC	WC balanced
*F*_0_ [Å^3^]	–3.308 × 10^−1^	–1.339 × 10^−1^
*F*_1_ [Å^2^]	4.037 × 10^−1^	–1.704 × 10^−1^
*F*_2_ [Å^3^]	–4.418 × 10^−1^	–2.663 × 10^−2^
γ_0_ [kcal/mol/Å^2^]	3.669 × 10^−2^	1.916 × 10^−2^
*g*_1_ [(kcal Å^2^)/mol/e^2^]	8.695 × 10^2^	1.385 × 10^2^
*g*_2_ [(kcal Å^6^)/mol/e^4^]	–4.127 × 10^4^	–6.384 × 10^4^
*c*_1_ [Å^5^/e^2^]	1.325 × 10^3^	–7.221 × 10^3^
*c*_2_ [Å^9^/e^4^]	–9.393 × 10^5^	–1.419 × 10^6^
ξ	1.578 × 10^−1^	7.952 × 10^−2^

**Table 4:** Explicit Physical Feature Sets of the New IWM-GB Models Compared to Several Other Solvation Models: “+” Signifies That the Specific Physics Feature Is Present Explicitly in the Model, “–” That It Is Not^a^

solvation model	dipole polarization	CHA (Ω_2_)	octupole (Ω_0_)	quadrupole (*Q*_2_)	optimal boundary	pol/nonpol coupling	RMSE vs Expt
							Train∥Test
IWM-GB WC	+	+	+	+	+	+	0.71∥0.95
IWM-GB NC	+	+	+	+	+	–	0.79∥0.87
TIP3P (explicit)	+	+	+	+	N/A	+	1.05∥1.08
CHA-GB	+	+	–	–	–	–	1.20∥1.25
standard GB	+	–	–	–	–	–	1.50∥1.70
standard PE	+	–	–	–	–	–	1.40∥1.60
optimal boundary PE	+	–	–	–	+	–	1.52∥1.30

a Shown are the corresponding RMSEs (kcal/mol) of the calculated HFEs vs the experimental ref (152) for the train (88) and test (85) sets of rigid small molecules from the FreeSolv 0.52 dataset. Two variants of IWM-GB are considered: with polar/non-polar coupling (WC) and without it (NC). The CHA-GB estimates use the parameters specified in ref (174). The Standard PE (MEAD,^40^ 0.1 Å grid) and Standard GB (GBNSR6^78^ with ALPB^173^ correction, 0.5 Å grid) estimates use Bondi radii,^179^ with Δ*G*_np_ = γ*(SASA). “Optimal Boundary PE” employs ZAP-9 radii^110^ in the PE calculations. In all cases, GAFF^180^ and AM1-BCC partial charges are used.

Comparing the proposed new models to common GB (GBNSR6^78^) and PE (MEAD^40^) implicit solvent models, over both standard (Bondi radii^179^) and optimized (ZAP-9 radii^110^) DBs, the gap in performance widens to approximately 50%, over the small molecule sets used here. In other words, the IWM-GB approach has reduced the error of the “parent” GB by about *a* factor of 2.

**Figure 3:**
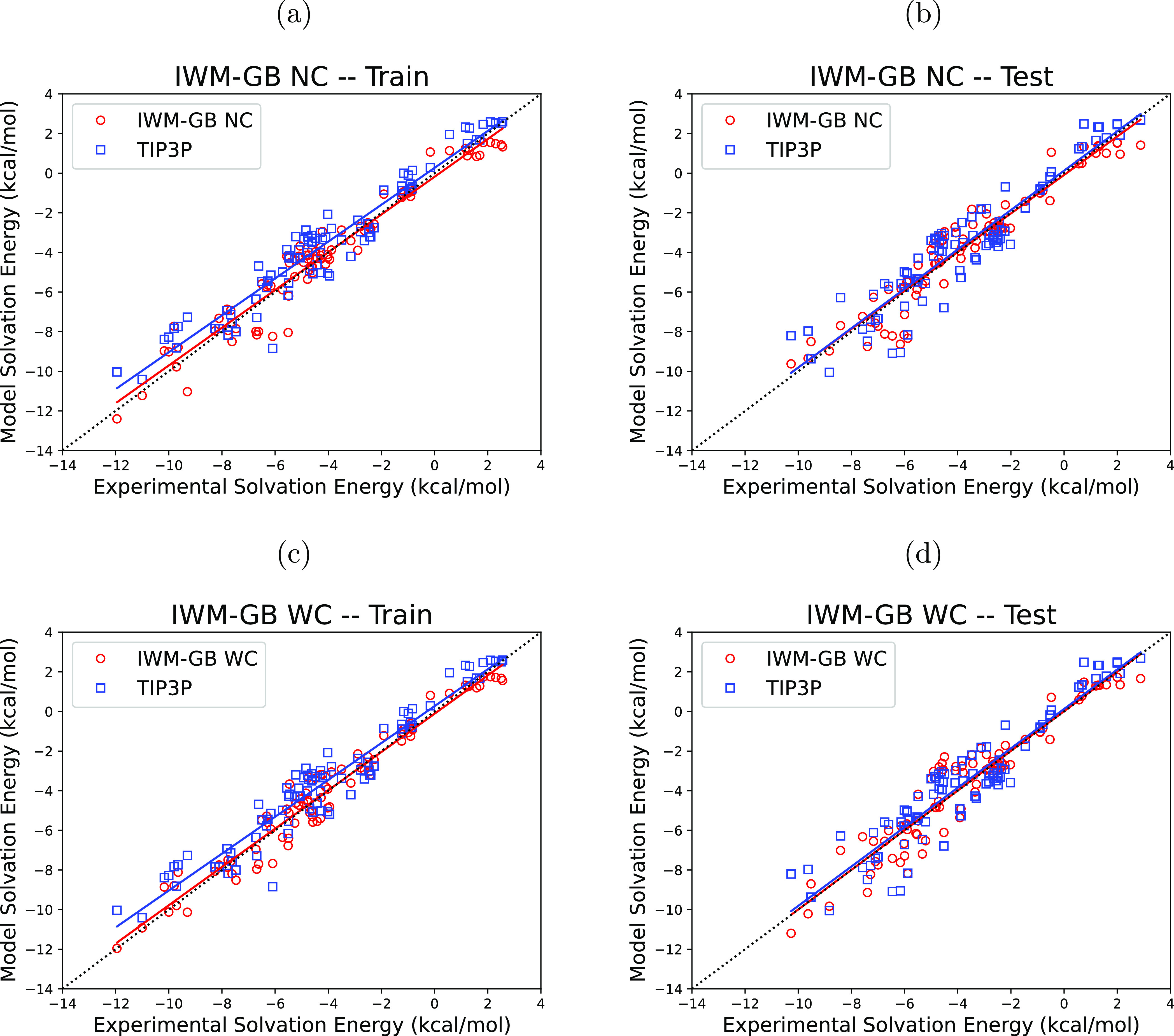
Comparison of the experimental and calculated hydration free energies for the optimized IWM-GB (implicit) and TIP3P (explicit) models of solvation (lines 1 to 3 of Table 4). For each panel, on both train (a,c) and test (b,d) sets, hydration free energies predicted by our proposed IWM-GB models WC and without it (NC, red circles) are plotted along with the corresponding explicit reference (TIP3P, blue squares), against experimental values.^152^ Linear best-fit lines are plotted for each considered model, with the same colors as individual hydration energies.

Figure 3 and Table 5 expand on each IWM-GB variant’s key performance metrics. In addition to improved RMSE values for both train and test sets, as compared to both explicit (TIP3P) and implicit (“Optimal Boundary PE”) reference models, *Mean Signed* and *Maximum Absolute* errors against experiment^152^ have been reduced too. The coefficient of determination, *R*^2^, has also improved for both train and test sets for both IWM-GB models. Interestingly enough, however, though the variant WC (IWM-GB WC), having one more optimized parameter, performs definitively better over the train set, it can only approximately match the NC variant (IWM-GB NC) over the test set. This may be indicative of some overfitting over the train set in the IWM-GB WC model.

**Table 5:** Error Statistics for the IWM-GB Models with Polar to Non-Polar Coupling (WC) and without the Coupling (NC), as well as the Explicit (TIP3P) and Implicit (“Optimal Boundary PE” Using ZAP-9 Radii^110^) References^a^

model	Train set				Test set			
	mean signed error	max absolute error	*R*^2^	RMSE vs experiment	mean signed error	max absolute error	*R*^2^	RMSE vs experiment
IWM-GB WC	–0.02	1.80	0.95	0.71	–0.01	2.30	0.90	0.95
IWM-GB NC	–0.01	2.53	0.94	0.79	–0.02	2.47	0.91	0.87
explicit TIP3P	–0.55	2.76	0.89	1.05	–0.15	2.89	0.88	1.08
“optimal boundary PE”	–0.19	5.20	0.80	1.52	0.00	3.13	0.85	1.30

a Mean signed error and maximum absolute errors, as well as HFE RMSEs versus experiment^152^ are given in kcal/mol, for both Train and Test sets. *R*^2^ is the respective coefficients of determination against the experimental HFEs.

We would also like to note that the usage of the DB surface instead of the SAS to calculate the contributions of the higher multipoles to the RF potential in eq 3 to compensate the omission of the integration over the whole solvent volume (discussed in the Section 2.2) provides 3–5% accuracy gains for both IWM-GB WC and IWM-GB NC models, over both the train and test sets.

### 3.2 Physics of the New Models

To the best of our knowledge, the IWM-GB models we have presented are new, so a deeper investigation of their physical properties is in order. In the previous sections, we have evaluated the performance of the models based only on the accuracy of the solvation free energies of small molecules. In particular, both the NC and WC variants of the IWM-GB model appear to have very similar accuracy compared to the experiment; does that mean that the introduction of the polar/nonpolar coupling in the WC variant has no effect? We will address this, and several other questions below, which may help to reveal hidden drawbacks and suggest avenues for improvement. All of the following analyses are performed on the test set data only.

#### 3.2.1 Relative Multipole Contributions

Within each new optimized IWM-GB model, we further examine the contributions to the hydration energies of the multipole correction terms in eq 3 (as mentioned above, we use the mathematically equivalent eq 4 in our actual estimates).^e^ As a percentage of the “pure PE/GB” dipole energy, equivalent to the contribution of the first term in eq 3, the average *total* magnitude of the multipole corrections (the combined contributions of the remaining terms) is ∼14% for the IWM-GB WC model, not exceeding 48% for any of the molecules in the set. For the IWM-GB NC model, the corresponding average *total* multipole correction magnitude is ∼68%, not exceeding 132% for any of the small molecules.

A more detailed analysis is shown in Table 6, which presents the average relative contributions of different multipole terms (relative to the dipole term contribution) for the two optimized IWM-GB models. One can see that, in contrast to the IWM-GB NC model, the IWM-GB WC model has much more balanced separate contributions from the quadrupole and octupole correction terms, each of which not exceeding (in magnitude) the contribution of the dipole term. Moreover, the contribution of any of the octupole terms does not exceed that of the quadrupole terms. The smaller quadrupole and linear octupole term contributions in the IWM-GB WC model are mostly due to 2–3 times smaller magnitudes of the quadrupole (*F*_1_) and linear octupole (*F*_2_) parameters compared to the corresponding parameters in the IWM-GB NC model, see Table 3. This relative ordering of the multipole contributions in the IWM-GB WC model, in which higher order terms tend to contribute less to the total, is consistent with what is expected for a multipole expansion of hydration energy. This physically expected behavior is distinctly absent from the IWM-GB NC model; the contributions of the higher order terms are relatively large. But, being of the opposite signs, these mostly cancel each other, resulting in the smaller total multipole correction magnitude (Table 6, last column) and, perhaps somewhat deceptively, very reasonable total solvation free energies, Table 5. Thus, even though the RMSEs to experiment of the optimized IWM-GB WC and NC models are very close, Tables 5, the WC model appears to stand on a more solid physical ground compared to the NC variant, providing an argument for the need for the polar to nonpolar coupling.

**Table 6:** Average Fractions of the Multipole Corrections due to Quadrupole, Cubic Octupole, and Linear Octupole Terms Relative to the Dipole Term Contribution for the Two IWM-GB Models

model	quadrupole	cubic octupole	linear octupole	total correction
IWM-GB WC	0.48	–0.14	–0.48	–0.14
IWM-GB NC	–3.16	–0.06	2.54	–0.68

Further differences between the WC vs NC variants come in the form of their respective optimized atomic radii, Table 2. One can see that for both variants of the IWM-GB model, the respective atomic radii vary in both magnitude and relative orderings, as compared directly to Bondi and ZAP-9 radii sets. The significantly larger hydrogen and the slightly larger oxygen and carbon atomic radii of the WC model lead to a decrease of the dipole level polar energy contributions and the van der Waals portion of nonpolar energy contributions compared to the NC model. The discussion on notable characteristics of WC and NC variants of the IWM-GB model, with respect to the model’s nonpolar contribution to HFE is continued in Section 3.2.3.

#### 3.2.2 Charge Hydration Asymmetry

One of the unique features of water as a solvent, which can be used to characterize the quality of explicit and implicit solvation models,^157^ is the so-called CHA effect,^90−101^ which is strong in real water. The most prominent manifestation of the CHA effect is the difference in experimental HFEs of oppositely charged ions of similar size,^90,182^ such as K^+^ and F^–^.

For neutral solutes, a quantitative way to characterize the ability of a solvation model to describe the experimentally observed CHA effect is by evaluating, and comparing with experiment, the hydration energies of the special pairs of molecules called “CHA-conjugate”^157^ pairs of molecules. These pairs serve as analogues of ion pairs such as K^+^/F^–^: the split of hydration energies between these “CHA-conjugate” molecules, called cation-like (*B*^(+)^) and anion-like (*A*^(−)^) molecules,^157^ is qualitatively equivalent to the hydration energy split seen in a pair of ions of the opposite charge but of the same physical size. This split, ΔΔ*G* = Δ*G*(*B*^(+)^) – Δ*G*(*A*^(−)^), can be characterized by parameter^98,157^ η* = |ΔΔ*G*/⟨Δ*G*⟩|, where ⟨Δ*G*⟩ is the average hydration energy of the two molecules making up the “CHA-conjugate” pair.

In the case of the IWM-GB NC model, η_NC_^*^ = 0.09 for the pair 3-methyl-butanoic-acid (*B*^(+)^) and *N*-methyl-acetamide (*A*^(−)^). This value is small compared to η_exp_^*^ = 0.49, derived from the experimental hydration energies of the pair or η_TIP3P_^*^ = 0.43^157^ for the TIP3P water model. On the other hand, in the case of IWM-GB WC model, η_WC_^*^ = 0.28, providing a much closer to reality description of the hydration energies, making it an additional argument in favor of the IWM-GB WC model over IWM-GB NC model.

#### 3.2.3 Nonpolar Energy Contributions

**Figure 4:**
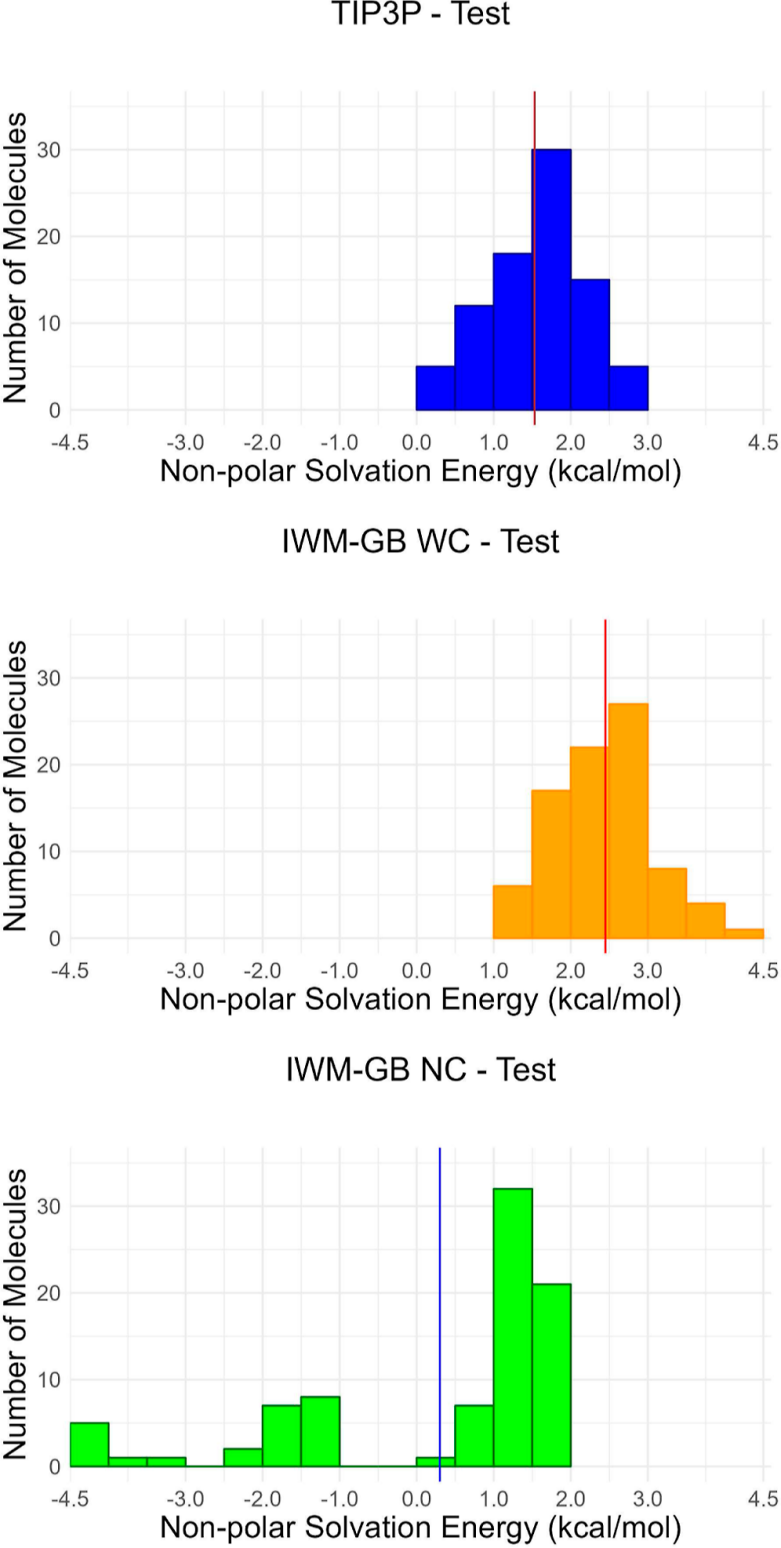
Comparison of the nonpolar energy distributions for the TIP3P (explicit) and our proposed IWM-GB WC and IWM-GB NC implicit solvent models on the test set of small molecules. Solid vertical lines indicate positions of the average values for each distribution.

An analysis of the nonpolar components (Δ*G*_np_) of the two optimized IWM-GB models and comparison with the corresponding TIP3P values provides some additional insights. The range of the TIP3P nonpolar energies is from 0.05 to 2.75 kclal/mol, with an average of 1.53 kcal/mol. While TIP3P is also just a model, the fact that it yields a positive nonpolar component of the HFE makes physical sense: this is the free energy cost of solvating a completely uncharged “oil droplet” in the shape of the original molecule. The nonpolar energies from the IWM-GB WC model have the same property that makes physical sense: the range of the Δ*G*_np_ values for this model is from 1.06 to 4.21 kcal/mol, with an average of 2.45 kcal/mol. In contrast, the corresponding values for the IWM-GB NC model can be strongly negative, ranging from −4.33 to 1.88 kcal/mol, with an average of 0.30 kcal/mol. Figure 4 shows the distributions of the nonpolar energies for each of these three water models, illustrating the similarity of the IWM-GB WC and TIP3P models and their differences from the IWM-GB NC model.

The exclusively positive Δ*G*_np_ values for the IWM-GB WC model suggest that the cavity term, that is the first term in eq 6, always prevails over the always negative van der Waals term.^31,153,163^ The presence of strongly negative Δ*G*_np_ values in the distribution for the IWM-GB NC model and the smaller average value suggest nonphysically large contributions of the negative vdW terms. In terms of the model parameters, we attribute the negative Δ*G*_np_ of the NC model to the relatively small atomic radius for “H” atoms (at roughly the same size as the water probe, see Table 2), and to the relatively large scaling coefficient ξ_N_ for “N” atoms, see Table 3.

In summary, multiple metrics suggest that the IWM-GB WC model, in which a coupling between the polar and nonpolar contributions is explicitly present, is distinctly more physically sound than the version in which such a coupling is absent.

### 3.3 Toward a Well-Balanced Model

The IWM-GB models presented in Tables 4 and 5, and Figure 3 were optimized for a subset of rigid small neutral molecules; the accuracy of these models well outside of the subset may deteriorate. These would include molecules with substantially more negative HFEs than those seen in Figure 3, and especially charged molecules. In addition, our calculations so far have relied on the atomic partial charges used in the General AMBER Force Field^180^ (GAFF) and generated by the AM1-BCC method.^183^ Applying these optimized IWM-GB models to molecules described by other sets of FF parameters may lead to worse accuracy.

In particular, the accuracy of the IWM-GB models in the critically important case of amino acids (AA), which utilize a slightly different set of atomic partial charges (RESP^184^) and FF,^185^ is not guaranteed to be as good as seen for the subset of small molecules in Tables 4 and 5. While experimental HFEs for the 20 essential AA are unavailable, and it is unclear how these could even be measured for the charged ones, one can use^150^ TIP3P polar solvation energies, Δ*G*_el_, as a reasonable proxy to assess the overall performance of the new IWM-GB models for this critically important class of small molecules. A test of the two optimized IWM-GB models, presented in Table 4, against TIP3P polar hydration energies of 22 blocked AA^109,174^ (including neutral forms of Glu and Asp^174^) yields fairly large RMSEs: 5.39 kcal/mol for the IWM-GB WC model and 6.34 kcal/mol for the IWM-GB NC model.

Looking at these two numbers, it is worth noting that the IWM-GB WC model, in which an explicit coupling between polar and nonpolar parts of the solvation free energy is introduced, produces a smaller RMSE compared to the NC variant. Thus, the model with more correct physics taken into account—the dependence of the SAS position on the strength of a solute local electric field—produces more realistic outcomes well outside of the train set. Still, even this lower RMSE for the WC variant appears too large for the critical case of AA, most likely due to some of the reasons mentioned above.

To address the issue, we have reranked the IWM-GB WC model optimization outcomes with an objective function that combines RMSE for AA with the same weight as for the small molecules. The lowest combined RMSE model resulting from this procedure is the “balanced IWM-GB WC” model, which can be considered a reasonable compromise, well-balanced solution. This “balanced IWM-GB WC” model has low RMSE values relative to the experiment for the small molecule sets, see Table 7, and also a reasonable RMSE of 1.92 kcal/mol for Δ*G*_el_ of the blocked AA, relative to the TIP3P polar refs (109 and 174). The radius of the sulfur (S) atom, present in two AA (Met and Cys), is taken directly from the ref (174) (includes an *R*_s_ correction for the DB position), where atomic radii were optimized for CHA-GB model. This “balanced” solution is CHA-compliant, η* = 0.14, and can be used simultaneously for the systems containing both AAs (proteins) and small molecules.

**Table 7:** Error Statistics for the “Balanced” IWM-GB WC Model^a^

model	Train set of small molecules				Test set of small molecules			
	mean signed error	max absolute error	*R*^2^	RMSE vs experiment	mean signed error	max absolute error	*R*^2^	RMSE vs experiment
IWM-GB WC balanced	–0.00	3.38	0.92	0.90	–0.09	2.69	0.90	0.95

a Mean signed and maximum absolute errors, as well as HFE RMSE values versus experiment are given in kcal/mol. *R*^2^ is the respective coefficient of determination against the experimental HFEs.

**Figure 5:**
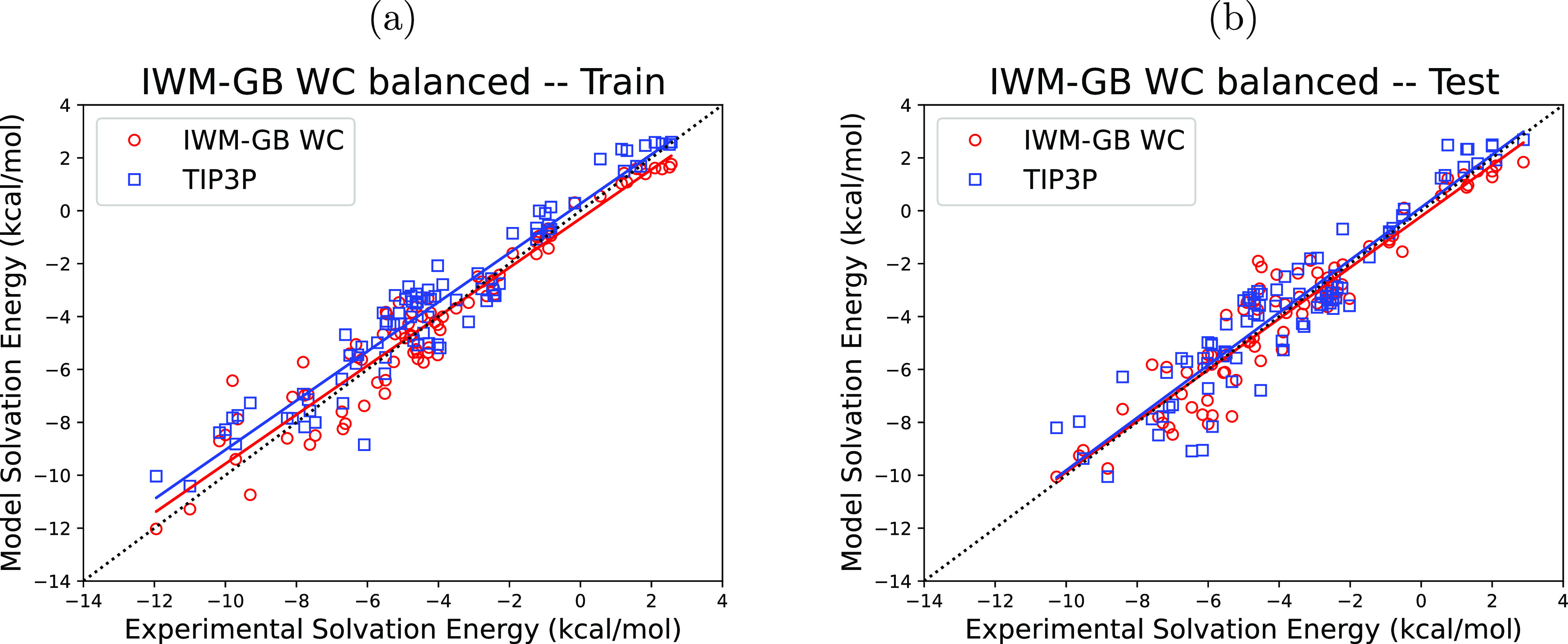
Comparison of the experimental and predicted hydration free energies of small molecules for the “balanced” IWM-GB WC solvation model. For both train (a) and test (b) sets, hydration free energies of the proposed “balanced” IWM-GB WC model (red circles) are plotted along with the corresponding TIP3P explicit water model data (blue squares), against the experimental values. Linear best-fit lines are plotted for each considered model, with the same colors as individual hydration free energies.

Figure 5 and Table 7 present key accuracy metrics for our balanced IWM-GB WC model. Compared to the TIP3P explicit solvent model performance (see Table 5), the balanced IWM-GB WC model has smaller RMSE to experiment values on both train and test sets of small molecules, a slightly better coefficient of determination, *R*^2^, and much smaller mean signed errors. Relatively small difference between the train and test set RMSEs suggests that there is no substantial overfitting over the train set in this “balanced” model. The average magnitudes of the total multipole corrections to Δ*G*_el_ relative to the dipole level contributions [Δ*G*_el_^dipole^, as before, calculated using the GB model (eq 9)] are ∼32% for the train and ∼34% for the test sets of small molecules, not exceeding 61% for any of the molecules in these sets.

## 4 Conclusions

We have proposed a relatively efficient implicit solvation framework—Implicit Water Multipole GB (IWM-GB) model—that incorporates multipole moments of water molecules and the corresponding polarizations of water, in addition to the dipole polarization already present in the PE or the GB models of solvation. Here, we take the PE/GB level of theory as the “ground state”, and add to it the contributions of the quadrupole and octupole polarizations in the first hydration shell of a solute as systematic corrections to the solvation energy. The proposed framework also accounts for, in a computationally efficient manner, the coupling between polar and nonpolar contributions to the total solvation energy, present in reality, but missing from many practical implicit solvation models.

A pilot implementation of the framework reported here uses a version of the GB model, GBNSR6, known to closely approximate the PE treatment in computing the HFEs of molecules at a fraction of the computational cost.^186^ We use GAFF and AM1-BCC atomic partial charges. Key adjustable parameters of our IWM-GB models include five atomic and water probe radii, and the relative weights of the multipole contributions to the electrostatic solvation free energy; another set of parameters describes the nonpolar contributions and the polar/nonpolar coupling. The parameters of IWM-GB are optimized for best agreement with the experimental HFEs of a subset of small molecules from the FreeSolv database.^152,175^ The solutions are tested against a different subset of small molecules from the same database and blocked AA analogues. The cost of adding the extra multipole terms to the dipole (GB) level energy for our unoptimized pilot implementation of IWM-GB models is about 10-fold of the GB estimate alone; based on the computational complexity of the numerical terms involved, we argue that the cost can be reduced to about 3-fold of the GB.

Two versions of our IWM-GB models are investigated in detail. One of these versions, IWM-GB WC, includes the polar/nonpolar coupling explicitly, via additional terms that depend on the distribution of partial charges in a solute, while the “no coupling” variant (IWM-GB NC) uses standard decomposition in which the polar and nonpolar parts of the solvation energy are computed independently of each other.

Our first main conclusion, based on several common accuracy metrics evaluated on the test set against experimental HFEs, is that the proposed inclusion of the multipole moments beyond the dipole improves the resulting accuracy appreciably. The overall accuracy of both versions of IWM-GB is higher than that of the TIP3P model, with a 12% reduction of the RMSE relative to the experiment. Compared to the baseline GB treatment, the RMSE of IWM-GB decreases by about 2-fold.

While the accuracy of the two variants of the model—with (IWM-GB WC) and without (IWM-GB NC) coupling between the polar and nonpolar contributions—is very similar, a more detailed analysis of the physical properties of these models reveals significant differences between them. Specifically, the WC variant exhibits only non-negative nonpolar contributions for all of the molecules, while the absence of the coupling leads to highly negative, nonphysical nonpolar components for a number of molecules. Also, in the WC model, the successive contributions from the multipole moments are smaller than the dominant dipole contribution, in stark contrast to what is observed for the NC model, where these contributions can be as high as three times that of the dipole term. The charge hydration asymmetry (CHA) effect is also close to experimental expectations in the WC model. The above observations lead to our second main conclusion: the polar to nonpolar coupling is necessary to ensure that the model makes physical sense, not merely reproduces experimental reference with good precision, which may be the result of an error cancellation between large individual contributions.

The proposed IWM-GB framework has several limitations. First, not all of the highly nuanced physics of hydration are included in it explicitly. Specifically, water molecules are known to sometimes form “water bridges” (hydrogen bonds) between two atoms in polypeptides;^111,187−194^ these are not considered in the current IWM-GB models. Solute charges attracting nearby water molecules can cause an increase of the water dipole density (electrostriction) and related water polarization. Also, in a strong enough solute electric field, the linear response approximation breaks down, becoming more sublinear (dielectric saturation). These two effects, not accounted for in the linear response PE/GB framework, may cancel each other out^195^ to an extent; in the future, one may consider including the remaining difference^196^ directly into the model. Electronic polarizability of water molecules,^197−200^ as well as charge transfer effects are also not considered explicitly. We note, however, that these effects are included implicitly, albeit in an average sense, via the parameters of the IWM-GB models.

Our IWM-GB models have not been parametrized separately against AA solvation energies, however, some of the model parametrizations (the “balanced” IWM-GB WC model) show good RMSEs both for small molecules and also for blocked AAs, including the charged ones. This is suggestive that the model might perform reasonably well for proteins, but we stress that we have not tested it yet in this context. At the moment, our new optimized IWM-GB models can only be applied to molecules that contain H, O, N, C, and S atoms only (for sulfur, the models have only been tested for AAs). Inclusion of other atom types, such as I, F, Br, and Cl, will require additional rounds of optimization. Operationally, the proposed framework can be used with numerical PB solvers^39−42,201^ instead of the GB, but that option has not been tested yet, and may require fine-tuning of the model parameters.

In summary, a novel implicit solvation framework is proposed; it shows promise, while highlighting the need to account for subtle solvation effects absent from most practical implicit solvation models. Our ultimate goal is to create an efficient implicit solvation model that describes all types of molecules equally well, within the same model and with the same set of parameters. The general approach proposed in this work may be the first (but certainly not the last) step in this direction.

## Footnotes

a If the model parameters are fitted against reference training data containing the coupling, or any other physical effect for that matter, the effect may still may be present in the model implicitly, but only in an average sense; the correct physical response function is lost.

b While the DB can be easily described in intuitive terms as “a boundary separating the solute and the solvent”, the details matter; there is more than one way^118,154,155^ of rigorously defining and computing the DB. The definition used in this work is discussed in detail below.

c In what follows, the water multipoles are also placed at the DB.

d We have also tested the robustness of the IWM-GB models to the choice of the gas-phase FF. Repeating the same optimization procedure with GAFF2 parameters,^181^ including the corresponding partial charges, has led to essentially the same RMSE values for the WC and NC models, as reported in Table 4. Namely, for the IWM-GB WC model, the train and test set RMSEs are 0.81 and 0.91 kcal/mol, respectively; for the IWM-GB NC model, the corresponding numbers are 0.90 and 0.89 kcal/mol, respectively.

e In estimating these contributions, we excluded several molecules with very small, less than 0.1 kcal/mol, magnitudes of the dipole energy term to avoid numerical errors due to division by near zero values. Using a somewhat larger threshold has little effect on the computed fractions of the multipole contributions, since those few molecules with near zero dipole contributions are already excluded with the 0.1 kcal/mol threshold.
